# To Blame or to Forgive? Reconciling Punishment and Forgiveness in Criminal Justice

**DOI:** 10.1093/ojls/gqv012

**Published:** 2015-04-02

**Authors:** Nicola Lacey, Hanna Pickard

**Keywords:** punishment, retribution, criminal justice, ethics, political theory, forgiveness

## Abstract

What do you do when faced with wrongdoing—do you blame or do you forgive? Especially when confronted with offences that lie on the more severe end of the spectrum and cause terrible psychological or physical trauma or death, nothing can feel more natural than blame. Indeed, in the UK and the USA, increasingly vehement and righteous public expressions of blame and calls for vengeance have become commonplace; correspondingly, contemporary penal philosophy has witnessed a resurgence of the retributive tradition, in the modern form usually known as the ‘justice’ model. On the other hand, people can and routinely do forgive others, even in cases of severe crime. Evolutionary psychologists argue that both vengeance and forgiveness are universal human adaptations that have evolved as alternative responses to exploitation, and, crucially, strategies for reducing risk of re-offending. We are naturally endowed with both capacities: to blame and retaliate, or to forgive and seek to repair relations. Which should we choose? Drawing on evolutionary psychology, we offer an account of forgiveness and argue that the choice to blame, and not to forgive, is inconsistent with the political values of a broadly liberal society and can be instrumentally counter-productive to reducing the risk of future re-offending. We then sketch the shape of penal philosophy and criminal justice policy and practice with forgiveness in place as a guiding ideal.

## 1. Introduction

What do you do when faced with wrongdoing—do you blame or do you forgive? When confronted with crime, especially offences that lie on the more severe end of the spectrum and cause victims terrible psychological or physical trauma or death, nothing can feel more natural than blame. We may feel a range of hostile, negative emotions, such as hate, anger, resentment, indignation, disgust, contempt and scorn towards the perpetrator. We may judge them harshly, condemning their character. We may want them punished and to suffer in turn for what they have done. Moreover, we may feel entitled to these sorts of emotions and attitudes, as reactions which are *deserved* by the offender. Indeed, in the UK and the USA, increasingly vehement and righteous public expressions of blame and calls for vengeance have become commonplace in wider society.^1^ On the other hand, people can and routinely do forgive others, even in cases of severe crime.^2^ Evolutionary psychologists argue that both vengeance and forgiveness are universal human adaptations that have evolved as alternative responses to exploitation, and, crucially, strategies for reducing the risk of future re-offending.^3^ We are naturally endowed with both capacities: to blame and retaliate, or to forgive and seek to repair relations. We have a choice. Which should we choose?

Contemporary penal philosophy has witnessed a resurgence of the retributive tradition, in the modern form usually known as the ‘just deserts’ or ‘justice’ model.^4^ On this model, punishment is hard treatment visited on the offender in response to, by reason of, and in proportion to his or her ‘desert’ or blameworthiness. Blameworthiness, in turn, demands that the offender have the capacity for *responsible agency*: minimally, cognitive and volitional capacities such that they knew what they were doing when they committed the offence, and exercised choice and a sufficient degree of control in doing so. According to this tradition, punishment is only justified if the condition of responsible agency is met, and it is further limited by the requirement that it be proportional to blameworthiness.^5^ Hence our propensity for vengeance is to that extent tempered, rather than left wholly unchecked. Nonetheless, in forging a strong association between the justification of punishment and the appropriateness of blame, the choice made is clear—according to this model, when confronted with culpable wrongdoing, we should blame, not forgive.^6^

Our aim in this article is to explore the possibility that this choice—to blame, not forgive—which is garnering increasing consensus within penal philosophy, is inconsistent with the basic political values of a broadly liberal society, and stands in need of challenge. For these values demand that respect and equality ideally accrue to all. They therefore require responses to crime that aim—even if they do not always succeed—to restore offenders as full participant members of our society and repair the rupture to all that criminal offending creates. If indeed forgiveness functions to repair relations (while reducing the risk of future re-offending) then these values suggest that we ought—so far as possible—to replace blame with forgiveness as a guiding ideal within penal philosophy and criminal justice policy, and promote practices within criminal justice institutions that move away from blame and towards forgiveness within the vast spectrum of possible real-world responses.

This suggestion may at first glance appear so radical as to beggar belief. We hope that, as the article progresses, the contours of what it would mean, in theory and in practice, will become clear and credible. But it is worth stating at the outset that, in counselling forgiveness within penal philosophy and criminal justice policy and practice, we are not advocating *the abolition* of punishment, but rather its *reconception*. On the ‘justice’ model, punishment is the imposition of retaliatory *costs* or hard treatment in response to *blameworthiness*: it is an institutionalised form of blaming*.* We suggest that punishment be reconceived as an institutionalised form of forgiving: the imposition of *consequences* in response to *responsibility* for crime, enacted with forgiveness in that these consequences ought to be constructed, so far as possible, to embody reparative and corresponding risk-reduction strategies.^7^ In this respect, our proposal is allied with, yet distinct from, recent developments in restorative justice theory and practice. Like restorative justice, we aim to justify and develop criminal justice practices that move away from retribution, and towards reparation and rehabilitation. But unlike restorative justice, we do not suggest bringing victims and offenders together, with the aim of creating atonement in offenders and forgiveness in victims, as a means to this end. Rather, on our view, the criminal law itself can offer forgiveness, not on behalf or in place of victims, but in its own right. Similarly, criminal justice institutions and practices can be better designed to embody reparative and rehabilitative strategies. Hence, despite its radical appearance, our proposal aims to be pragmatic, suggesting how we can potentially shift cultural attitudes and reconfigure elements of existing criminal justice theory and practice, broadly speaking within the confines of the current system.^8^

The article proceeds as follows. We begin in section 2 by briefly re-visiting our argument, more fully articulated elsewhere,^9^ that there is both value and scope for importing to the criminal law a conceptual framework derived from clinical practice that sharply differentiates responsibility from blame. We use this framework to show how the law can operate with a robust notion of responsible agency as a condition of justifiable punishment, without thereby invoking the ‘worthiness’ or appropriateness of blame as a response to criminal wrongdoing. With this framework in place, we turn in section 3 to the nature of forgiveness. Drawing on both philosophical and legal traditions, we begin with an investigation of our ordinary folk psychological concept and the puzzle it is thought to create, before turning in section 4 to broaden the investigation, by drawing on evolutionary psychology to consider forgiveness in light of its possible function. To anticipate, we claim that we can understand forgiveness not simply as an intrinsic, subjective state of mind, but as one which additionally functions to motivate reparative behaviours. This allows us to draw on the nature of these behaviours to reconceive punishment as a response to responsibility for wrongdoing which imposes consequences—no doubt typically negative but occasionally not, so long as they are serious and appropriate to the crime and the context—*that embody reparative strategies*. In section 5, we flesh out the claim that we ought to replace blame with forgiveness as a guiding ideal within penal philosophy and criminal justice policy and practice, beginning with instrumental considerations, and moving to argue for this conclusion based on a commitment to the basic political values of a broadly liberal society. Finally in section 6 we briefly counsel against some tendencies within our current forgiveness practices that may undermine genuine forgiveness by in fact inviting blame back in to criminal law. And we explore what it could mean in practice to punish with forgiveness as opposed to punishing with blame, distinguishing various stages of the criminal justice process, including conviction, sentencing, and the execution and longer-term effects of the sentence.

## 2. Taking the Clinical Model of Responsibility Without Blame into the Legal Realm

In a previous article, we argued at length that there were compelling reasons to adopt the clinical model of responsibility without blame within penal philosophy and practice.^10^ We will not rehearse all the details of that argument here, but aim rather to provide the basic outline of the clinically-derived conceptual framework and illustrate its applicability to criminal law.

Within moral philosophy, as well arguably as the law and society at large, there is a deep-rooted tendency to link the idea of responsibility fundamentally to morality, by holding that its point or purpose is moral evaluation: the assessment of another and their behaviour as good or bad, right or wrong. In addition, such moral evaluation is often believed to be fundamentally affective in form—embodied and expressed in our emotions and reactive attitudes towards those whose actions show ill will towards others.^11^ These attitudes can include hate, anger, resentment, indignation, disgust, contempt and scorn, to reiterate the possibilities listed in the opening paragraph of this article, and of course are often accompanied by equally hostile expressions and actions. At its most radical, the link between responsibility and these attitudes is thought to be constitutive: ‘to regard oneself or another as responsible just is the proneness to react to them in these kinds of ways’.^12^ More modestly, to hold another responsible has instead been proposed to consist in believing that such reactions would be appropriate or fitting, even if one does not actually have the relevant feelings oneself.^13^ But such nuances aside, the idea of responsibility to emerge from this picture links it fundamentally to moral evaluation via our practice of responding to others with what is in effect an affective form of blame.^14^ Indeed, theories of criminal law that see the criminal process and the execution of punishment as a form of *institutionalised resentment* (alongside other attitudes) are often underpinned by this picture, with the law understood as functioning to uphold our common morality by assessing transgressions and condemning trangressors through institutional processes that stand proxy for our collective emotions and reactive attitudes towards wrongdoing.^15^

Clinical practice offers a very different model. Effective treatment of certain kinds of disorders of agency—where core symptoms or maintaining factors involve actions and omissions including, but by no means restricted to, those that cause harm to others or are wrong—may require clinicians to engage with patients as responsible agents with regard to their behaviour in order to help them to change.^16^ Such problematic behaviour is often a habitual if ineffective way of coping with psychological distress, and so part of a cycle of dysfunction. Improvement or recovery from disorders of agency therefore requires patients to break the cycle by doing things differently. Responsibility—understood, as in criminal law, as possession of minimal cognitive and volitional capacities—is essential for this to be possible. For it is only possible to deliberately and directly change behaviour over which we have choice and at least a degree of control.

Hence the clinical task with such patients is, in part, to motivate, encourage, and support them to take responsibility and do things differently. Importantly, this can involve asking patients to be accountable when they don’t. Although forms of accountability vary between therapeutic modalities, they can include challenging feedback, so that the negative effects of problematic behaviour on self, others and relationships is made explicit and must be faced, potentially alongside the imposition of negative consequences (usually with advance warning, and patient agreement). Although these consequences typically involve a reflective component to encourage patients’ understanding of why they lapsed on this occasion, and to develop a plan for how to succeed next time, they may also involve measures that potentially feel punitive, such as withdrawal of privileges, or time-limited suspension from a therapeutic group.

It is a staple of clinical practice that, because these forms of accountability potentially feel punitive, they must be enacted with an attitude of concern, respect and compassion for the patient, as opposed to being accompanied by or expressive of any of the hostile, negative emotions or reactive attitudes connected to affective blame. Within the clinic, responsibility is understood as fundamentally linked simply to agency, not morality: the point or purpose of the idea of responsibility and a demand for accountability is to enable and empower patients to change behaviour that is problematic for the sake of their wellbeing or recovery. When the problematic behaviour causes harm to others alongside its impact on patients themselves, clinicians will of course recognise this impact on others and the moral dimension of the behaviour. Correspondingly, they may form a judgment of ‘detached blame’, which attributes to patients responsibility for harm to others or wrongdoing.^17^ But the aim of clinical engagement is not to form such judgments or morally evaluate patients, but to care for patients and help them to change—irrespective of whether the problematic behaviour does or does not cause harm to others or is wrong. Affective blame is understood within clinical practice to undermine the capacity of responsibility and accountability to enable change, because of its propensity to make patients feel rejected, worthless, ashamed and uncared for, thereby rupturing the therapeutic relationship as well as damaging any sense of hope for the future, and, correspondingly, motivation and belief that they really can do things differently.

The clinic thus offers a corrective to the widespread tendency to link responsibility with affective blame by offering a clear and established practice of attributing responsibility for problematic behaviour—including morally problematic behaviour—and holding to account without affective blame, but instead with an attitude of concern, respect and compassion. Put crudely, reflection on clinical practice brings into sharp relief a distinction between whether *the patient* has choice and a sufficient degree of control over their behaviour to be appropriately asked to take responsibility and potentially held to account, and how *others respond* to patients when they are indeed responsible for behaviour—again, including morally problematic behaviour—and are being held to account. Patients may be responsible and held to account for behaving in ways which are harmful or wrong, yet clinicians engage and act without affective blame.

Although there are important differences between the clinic and criminal justice institutions, this clinically derived conceptual framework can yet be imported in many respects to the criminal law.^18^ In the law as in the clinic, responsibility is contingent on possession of cognitive and volitional capacities. Punishment—as a form of accountability—is only appropriate in relation to the degree of responsibility for and severity of an offence. But this fact does not in itself entail the ‘worthiness’ or justification of affective blame as a response to criminal wrongdoing any more than it is so entailed as a response to problematic behaviour—moral or otherwise—in the clinic. Hence the emphasis placed by the ‘justice’ model on degree of responsible agency and severity of offence as a condition and limit to justifiable punishment^19^ can be maintained wholly apart from the rhetoric of ‘just deserts’. Affective blame can be severed from punishment, thereby allowing for penal practices to be fashioned that better serve reparative and rehabilitative ends. Punishment can be understood not as hard treatment or the imposition of retaliatory costs in response to blameworthiness, but, similarly to the clinic, as the imposition of *consequences*—no doubt typically negative but occasionally not, so long as they are serious and appropriate to the crime and the context—in response to responsibility for crime.

Given that responsibility and accountability are maintained by this reconception of punishment as proceeding without affective blame, we have good reason in general to adopt it if indeed doing so better serves reparative and rehabilitative ends. What we aim to explore in this article is the possibility that these ends are best served not only by punishing *without affective blame*, but further, by punishing *with forgiveness*. In other words, given the choice to blame and to forgive, we should choose to replace blame with forgiveness as a guiding ideal within penal philosophy and criminal justice policy and practice. We therefore turn next to the task of articulating the nature of forgiveness and the reasons why, out of all the many positive sentiments that could be fostered within criminal justice processes that yet leave responsibility attributions and punishment intact, forgiveness is particularly well suited to play this role.

## 3. The Meaning of Forgiveness

Forgiveness is often allied with a range of emotions and reactive attitudes that express goodwill or positive regard, such as compassion, empathy, kindness, clemency and mercy.^20^ Within legal philosophy, mercy in particular has been singled out as valuable if not indeed essential to the justification of punishment.^21^ Our first step therefore is to distinguish forgiveness from mercy, as the two are easily confused.

On the one hand, the grounds for both mercy and forgiveness may converge. Both can stem from compassion and empathy, which may occur in response to offenders who ‘make good’ by expressing guilt, regret and remorse, and apologising and offering reparation.^22^ Note that these various attitudes and actions on the part of the offender are not excuses or justifications: in themselves they do not bear on degree of responsibility or severity of offence. But they may nonetheless function to obviate the felt need to inflict punishment, for they offer evidence that some of the (non-retaliatory) ends that we may hope punishment serves, such as reduction of risk of re-offending, or atonement and the making of amends, have already been secured without it. With the exception of theories such as Kant’s^23^ that punishment is not merely justified but *necessitated* by the ‘justice’ model’s conception of blameworthiness, consideration of these grounds may therefore incline us to show mercy for the offender or to forgive the offence, and therefore to punish less if at all. Hence, on the other hand, mercy and forgiveness may converge not only in their grounds but in their outcome: both may result in the voluntary withholding of punishment.

Yet mercy and forgiveness are distinct attitudes. Despite the fact that both can affect the decision of whether and to what extent to punish, once punishment has been determined and enacted, the question of mercy is otiose, while the question of forgiveness remains.^24^ There is no question of whether or not to be merciful if we are not in a position to punish, whether that is because we have punished already, or because, more simply, we lack the power to do so. Mercy is a possible sentiment only for those with power and authority, exercised necessarily *de haut en bas*.^25^ Yet there is a question of whether or not to forgive, wholly apart from our capacity to punish. The offender may have been appropriately punished and so paid their dues, and yet we find we do not forgive. Alternatively, we may find it in our hearts to forgive when no dues have been paid and we are not ourselves in any position to demand them, for we are powerless—unlike mercy, forgiveness can in theory be exercised *de bas en haut*, from powerless victim to powerful perpetrator.

There is ample reason for thinking that mercy should play a larger role in sentencing procedures than it typically does within contemporary courts.^26^ Many different kinds of consideration may be relevant to merciful sentencing, from compassion and empathy for those who come from impoverished backgrounds of poor opportunity, which is the case for many offenders,^27^ to concerns about the increasing numbers of people sentenced to prison in many countries, and about the length and quality of prison sentences.^28^ But, once the sentence is determined, there is nothing more mercy can do. In contrast, there is room for an attitude of forgiveness to shape the nature and execution of punishment—and its aftermath—in prison and in the community. We can hold offenders responsible and punish appropriately in relation to the offence, without vengeance and affective blame, but with forgiveness.^29^

What does this mean? Philosophical discussions of forgiveness typically puzzle over its very possibility. Forgiving is not forgetting, nor, alternatively, accepting or minimising the offence. To forgive, one must keep the wrongdoing of the offender clearly in view. But how can one both do this and yet forgive the offence? As Lucy Allais puts the puzzle: ‘Forgiving seems to mean ceasing to blame, but if blaming means holding the perpetrator responsible, then forgiveness requires *not* ceasing to blame, or else there be nothing to forgive.’^30^

The straightforward solution to this puzzle is that, as articulated in section 1 in relation to clinical practice, ‘blaming’ does not mean ‘holding the perpetrator responsible’, as the two can be kept clearly distinct. The offender may be responsible for wrongdoing and so ‘blameworthy’ to use the language of the ‘justice’ model: they knew what they were doing when they committed the offence and exercised choice and a sufficient degree of control in doing so. But the fact that they are responsible for wrongdoing and so ‘blameworthy’ does not entail that we must affectively blame them. Hence, once the distinction between responsibility and affective blame is properly recognised, the puzzle of forgiveness is dissolved: we can judge another responsible for wrongdoing and indeed hold to account and punish, without vengeance and affective blame, but with forgiveness. This is part of why an attitude of forgiveness—unlike, say, one of clemency or leniency—is particularly suitable to inject into criminal justice processes that keep responsibility attributions and punishment intact. There is still the task, however, of offering a positive account of what forgiveness is.

Many philosophical accounts of forgiveness hold that it involves overcoming the hostile, negative emotions that can comprise affective blame, in particular anger and resentment.^31^ Some further hold that forgiveness demands replacing hostile, negative emotions with goodwill or positive regard. But there are straightforward and well rehearsed objections to these views as standardly articulated. On the one hand, overcoming hostile, negative emotions can occur in the absence of forgiveness. The passage of time notoriously dissipates strong emotions even when one will not forgive. Alternatively, one may decide the offender is simply not worth the energy these emotions require, thereby overcoming them through a form of withdrawal and rejection, rather than through forgiveness. On the other hand, one can forgive a person for one offence, while still holding a further offence against them, and so not replace one’s hostile, negative emotions with goodwill or positive regard. Relatedly, one’s affective stance post-forgiveness may simply be one of neutral detachment: one is willing to forgive and let bygones be bygones, but that does not itself mean one feels positively disposed towards the person who has offended.

Lucy Allais has offered an account of forgiveness which aims to do justice to the intuition that forgiveness involves overcoming hostile, negative emotions, but meets these concerns.^32^ Allais holds that at its ‘heart’ forgiveness involves ‘wiping the slate clean’ in the sense of no longer holding a particular offence against the offender insofar as one forbears from allowing the offence to continue to affect one’s emotions and attitudes towards him or her as a person. This is a sophisticated version of the adage that we can hate the sin, but love the sinner. When we forgive, we can in one sense hate the sin, in that we keep the wrongdoing clearly in view and judge the offender responsible for it. But, although we may not love the sinner, we need not therefore hate them either. To put it in our terms, we need not affectively blame the offender for the wrongdoing that we judge them responsible for. Because of its attention to the detail of what it means to overcome hostile, negative emotions—namely, to judge another responsible for a particular offence, yet forbear from the hostile, negative emotions and attitudes towards them as a person that the offence initially engendered—Allais is able to meet the objection that not all ways of overcoming emotions count as forgiveness. One forbears from nothing in allowing time to dissipate strong feelings, and one does not forbear from hostile, negative emotions in the right way by overcoming them through withdrawal and rejection, for one yet allows the offence to colour one’s attitude to the person. Equally, her account allows that one can forgive an offender for one offence, while still failing to treat them with goodwill or positive regard, because one continues to judge them harshly for other reasons, or because one’s affective stance may rather be one of neutral detachment.^33^

We believe that Allais has articulated the ‘heart’ of forgiveness in many of its core instances. But there are two respects in which our understanding of the nature of forgiveness departs from hers.^34^ First, in keeping with many other accounts of forgiveness, Allais holds that only victims—or possibly those closely identified with victims—are in a position to forgive. Yet there is nothing in her account that demands this restriction. And the restriction strikes us as at odds with how we think of forgiveness within our society. For, it is not only the victims of offences who are prone to allow the offence to colour their emotions and attitudes towards the offender as a person—to affectively blame them. Third parties are also prone to do so. Why, then, when third parties stop blaming and instead forbear from allowing the offence to affect their emotions and attitudes towards the offender as a person, should that not count as forgiving the offender for the offence?^35^ Equally, forgiveness can also be a self-regarding attitude. We can also forgive—or fail to forgive—ourselves for our own wrongdoing.

The second respect in which we depart from Allais is that she seems to assume that one can only forgive *if* one antecedently affectively blames—in other words, if one initially experiences a range of hostile, negative emotions and attitudes towards the offender as a person engendered by the offence. But again, although this may accurately describe many core instances of forgiveness within personal relationships, we do not think an account of forgiveness requires this restriction. Forgiveness can be a sort of standing disposition as well as a particular act or event.^36^ When confronted with an offence, a person of forgiving disposition can forgive the offence not by *overcoming* their hostile, negative emotions and attitudes towards the offender as a person or *forbearing* from allowing these *to continue* to affect them in this way, but rather by *foreswearing* any and all hostile, negative emotions and attitudes that it would be natural to have towards the offender on the basis of the offence in the first place, while yet judging the offender responsible and accountable for the wrongdoing. Thus understood, forgiving an offender for an offence can forestall affective blame before it takes hold, rather than overcome it once it has.

These departures from Allais’ account are especially important for the possibility of a role for forgiveness within contemporary courtrooms and criminal justice institutions. Any such role requires a transposition of forgiveness from interpersonal relationships to institutional contexts. Some alternative versions of justice, such as restorative justice, bring victims and offenders together, with the aim of creating atonement in offenders and forgiveness in victims, using reconciliation between them as a means to restoration of the offender’s status within society.^37^ Such reconciliation can be powerful and important when it is achievable, but often it is not. Victims may be dead, or, alternatively, unwilling to participate in such a process, or unprepared to forgive, and we may rightly view it as wrong in such circumstances to demand participation and forgiveness from them. They are, after all, the victims of the offence. Moreover many offences (eg endangerment offences such as driving under the influence of alcohol, inchoate offences such as conspiracy, and offences of possession) are ‘victimless’, while others (eg sophisticated forms of fraud) involve victims who are unaware of their victimisation. Yet, as we discuss in section 4, there may be compelling reasons to believe that offenders should have the opportunity to be forgiven for their offence. Just as it is possible to see the criminal process and the execution of punishment as a form of institutionalised resentment, so too it is possible to see it as offering institutionalised forgiveness. This is what the law, as an institutional third-party, can offer: punishment with forgiveness, insofar as, from the perspective of the law and what it represents within our society, the slate is wiped clean.^38^

This may seem an especially obvious role for the law in relation to those offenders whose sentence has been served and so have ‘paid their dues’. Yet, even then, there are many respects in which contemporary criminal justice procedures are non-forgiving and create enduring forms of stigma. For instance, under the UK Rehabilitation of Offenders Act 1974, although convictions punished by a non-custodial sentence, or a prison sentence of less than two and a half years, are ‘spent’ some years after that sentence is served, convictions punished by a prison sentence of more than two and a half years remain on the person’s record for the rest of their life. Furthermore, all offenders retain obligations to disclose even spent convictions, for all kinds of criminal offence, when applying for positions in numerous professions, including medicine, education, social work, accountancy and law. In some jurisdictions, notably in many parts of the United States, a host of stigmatising disqualifications, such as ineligibility to vote, to participate in a range of occupations, and to receive a variety of benefits, persist for some time after the sentence has been served, and, sometimes, for life.^39^ Replacing such non-forgiving practices with practices that offer a clean—or at least cleaner—slate once the sentence has been served is arguably extremely important, not only in order to promote rehabilitation and restoration, but also for the sake of justice.

But, we argue, the law can do more than simply offer forgiveness to those who have paid their dues by amending such stigmatising procedures. In order to genuinely punish *with* forgiveness, and without vengeance and affective blame, the law must not first punish with these but then overcome them through forgiveness. This is no reconception of punishment. Rather, such an approach simply reverts to the conception of punishment as hard treatment and imposition of retaliatory costs that is characteristic of ‘just deserts’ theories, but with forgiveness added on in its aftermath. We suggest that the task for criminal justice is not to punish and then to forgive—to inflict hard treatment as a supposed means to fostering inclusion and reparation, as some ‘just deserts’ theorists, perhaps most notably Antony Duff, would argue.^40^ Rather, we suggest it is to punish with forgiveness—to *foreswear* vengeance and affective blame *in* punishing, not just afterwards. For this is what allows the reconception of punishment as the imposition of *consequences*—as opposed to hard treatment or retaliatory costs—in response to responsibility for wrongdoing to take hold, and for these consequences to be effectively fashioned to embody reparative and corresponding risk-reduction strategies. The possibility of reparation and rehabilitation can then in principle proceed in tandem with punishment—if, that is, it is reconceived as inflicted not out of vengeance and affective blame, but with forgiveness.

In the next section, we say more about what this means in theory. In section 5, we discuss the instrumental and ethical reasons we have to adopt such a reconception of punishment, and we then in section 6 briefly sketch some of the ways it could be implemented in practice within criminal justice policy and procedures.

## 4. The Evolutionary Psychology of Forgiveness

Thus far, drawing on legal and philosophical traditions, we have suggested that we can understand forgiveness as *foreswearing* any and all hostile, negative emotions and attitudes that it is natural to have towards the offender as a person on the basis of the offence. This is a precise, but negative, analysis of forgiveness: we know what forgiveness *does not* involve—affectively blaming the offender—but not yet what it *does*. To answer this question, in this section we broaden the investigation by drawing on evolutionary psychology to consider forgiveness in light of its possible function.^41^

Evolutionary psychologists argue that both vengeance and forgiveness are universal human adaptations that have evolved as alternative responses to exploitation and, crucially, strategies for reducing the risk of future re-offending.^42^ Exploitation is likely to have been a major adaptive problem that we faced during our evolution as a species. But it can stem from either in-group or out-group members, predicting different responses. Out-group members typically have limited fitness (eg survival and reproductive) or other forms of value to us for the simple reason that they are not part of our group. We do not have ongoing and potentially mutually beneficial relationships with them. From an evolutionary perspective, there is unlikely to be any inherent cost to killing or permanently incapacitating out-group exploiters.^43^ In contrast, in-group members may have substantial fitness or other forms of value to us, because we stand in ongoing and potentially mutually beneficial relationships with them. Killing or permanently incapacitating in-group exploiters therefore carries an inherent cost: the loss of a valuable or potentially valuable person or relationship. For this reason, in-group exploitation ideally requires adaptive strategies that protect against risk of future exploitation, while yet preserving the possibility of an ongoing mutually beneficial relationship. Both vengeance and forgiveness may have evolved as competing strategies with this function.^44^

The logic of vengeance is in effect the logic of deterrence. Vengeance involves hostile, negative emotions and functions to motivate aggressive and cost-inflicting behaviours. By threatening or imposing retaliatory costs, we signal that the expected benefits of exploitation must be adjusted against them, thereby protecting against future exploitation by decreasing the exploiter’s motivation to exploit. There are, however, two risks inherent in this strategy. First, responding to exploitation with hostile, negative emotions and threatening or imposing retaliatory costs risks creating a cycle of vengeance, whereby the exploiter then seeks vengeance in turn, and the possibility of preserving an ongoing mutually beneficial relationship is lost, as aggression escalates on both sides. In effect, vengeance risks rupturing relationships to such an extent that in-group members effectively become like out-group members—turning from possible friends to permanent foes, with no fitness or other forms of value attached to the relationship whatsoever. Second, retaliation can function to protect against risk of future exploitation only insofar as the exploiter genuinely fears getting caught and subjected to cost-inflicting behaviours: it fosters no intrinsic desire to end hostilities on the part of the exploiter—indeed, as just noted, it may increase it. The success of retaliation as a response to protect against risk of future exploitation therefore depends on adequate and ongoing monitoring of the exploiter and possessing and maintaining the power to effectively harm them in turn.

Forgiveness, in contrast, functions to motivate reparative behaviours, directing resources away from hostile, negative emotions and aggressive, cost-inflicting behaviours. Reparative behaviours include (at least) the following three types.^45^ First, the forgiver may communicate—in a non-aggressive and non-retaliatory way—to the exploiter the extent of the damage they have suffered because of the exploitation. Some costs are imposed inadvertently or at least without full knowledge and intention: when this is so, then the risk of future exploitation may be reduced if the exploiter learns the true consequences of exploitation for the exploited party. Second, the forgiver may indicate that, despite the damage, the possibility for a mutually beneficial future relationship exists if the exploiter commits to refraining from future exploitation—in effect, the forgiver will wipe the slate clean, if they can be convinced that the exploiter really will refrain from harming them further. Third, the forgiver may remind the exploiter of the mutually beneficial past relationship, in order to make salient the potential long-term costs to the exploiter of a permanent rupture to that relationship. By motivating reparative behaviours, forgiveness thereby functions to protect against risk of future exploitation by preserving the possibility of an ongoing mutually beneficial relationship and re-establishing or indeed increasing the perceived value of that relationship to the exploiter. The success of forgiveness as a risk-reduction strategy does not therefore depend on adequate and ongoing monitoring of the exploiter and possession of the power to effectively harm them in future—rather, it aims to foster an intrinsic desire to end hostilities on the part of the exploiter, by eliciting recognition of the damage to, and value of, the exploited party and their relationship with them. In effect, forgiveness is both forward-looking and genuinely conciliatory.

The most significant risk inherent in forgiveness as a risk-reduction strategy is that the forgiver is deceived by the exploiter into believing that the exploiter is committed to refraining from exploitation in future, and so is vulnerable in virtue of trusting the exploiter and continuing the relationship. This is part of why credible expressions of guilt, regret and remorse, apologies and the offering of reparation, and public declarations of future intention to refrain from future exploitation, facilitate forgiveness:^46^ they both indicate—but may also in addition help to create and establish through declared commitment and resolve—the value the exploiter now places on the exploited party. But equally, no doubt, the offering of forgiveness and the opportunity for a mutually beneficial relationship in future may serve to facilitate these responses on the part of the exploiter. Forgiveness is, in effect, a co-operative strategy: it works to benefit both parties if and only if both parties can be relied on to do their part—the exploited party must wipe the slate clean, and the exploiting party must forbear from further exploitation.

From an evolutionary perspective, reparation as opposed to retaliation is optimal when successful, for it reduces the risk of the exploiter perpetrating future harm, without incurring the costs of monitoring and maintaining the power to retaliate, and while bringing the benefit of preserving relationships so far as possible. Apart from context-specific elements, both individual and genetic differences (such as sex) appear to contribute to whether an individual opts for an avenging or forgiving strategy in response to exploitation.^47^ But a key factor in choice of strategy is ‘Associational Value’: the expected future value of the exploiter to the individual or group across a variety of domains, and as indicated by factors such as kinship, capacity for work or other forms of social productivity, level or remorse or repentance post-exploitation, and various other forms of mutually interdependent and benefit-conferring relations. Where Associational Value is high, the orientation to forgiveness and reparation is accordingly enhanced; where it is low, the orientation to retaliation will be stronger.^48^ Indeed, a series of striking experiments suggests that it governs intuitions about the appropriateness of punitive versus reparative responses to criminal wrongdoing in contemporary contexts.

Petersen and others conducted a series of studies^49^ both in the US and in Denmark that—despite clear differences in large-scale national attitudes towards crime, with Americans inclining towards punishment and Danes focusing on rehabilitation—converge on the same set of results. Subjects’ views of the severity of a crime predicted their view of the intensity of the response that was appropriate: the more severe the crime, the more intense the response needed to be. But their view of the severity of a crime did not predict their view of the kind of response that was appropriate: retaliatory or reparative/rehabilitative. The choice between a retaliatory or reparative/rehabilitative response was predicted instead by the Associational Value of the offender. Hence, independently of perception of crime severity, subjects preferred a retaliatory response for criminals with a low Associational Value, indicated by persistent offending, out-group status, or low levels of remorse, and a reparative/rehabilitative response for criminals with a high Associational Value, indicated by first-time offending, in-group status, or high levels of remorse. These results strongly suggest that Associational Value predicts the kind of response deemed appropriate. Severity of crime predicts, in contrast, the intensity of *either* kind of response deemed appropriate—worse crimes demand more retaliation or more reparation/rehabilitation, but do not demand retaliation as opposed to reparation/rehabilitation.^50^

These findings have striking implications. On the one hand, they demonstrate that the intuition that responses to crime must be ‘fitting’ or appropriate in relation to the degree that an offender is responsible in combination with the severity of the offence is not exclusive to the ‘justice’ model. Our basic intuition is that a severe crime requires an intense response, whether this is retaliatory or reparative/rehabilitative. This makes sense: where an offence is serious, on a vengeance strategy, there is more wrongdoing to punish, while on a forgiveness strategy, there is more reparative and rehabilitative work to be done. On the other hand, these studies suggest that, however natural vengeance may feel, forgiveness comes just as instinctively to our species, even in response to severe offences. We stand prepared to forgive—in that we prefer reparative/rehabilitative to retaliatory risk-reduction strategies—when we view the offender as a person of value to us and as one who, if they play their part in the forgiveness strategy we are prepared to offer, is worth trusting to value us in turn.

How then does drawing on evolutionary psychology allow us to offer a more positive analysis of forgiveness, over and above the negative claim that it requires forbearing from affective blame? The key idea that we want to emphasise is that both vengeance and forgiveness are not simply intrinsic, subjective states of mind, but can be viewed in functional terms. Both respond to the same input: exploitation. Both can be seen to serve the same end: reduction of risk of future exploitation, by decreasing motivation to exploit. Vengeance does this by signalling that the expected benefits of exploitation must be adjusted against retaliatory costs. Forgiveness does this by preserving the possibility of an ongoing mutually beneficial relationship and re-establishing or indeed increasing the perceived value of that relationship to the exploiter. But, correspondingly, vengeance and forgiveness have different outputs, consonant with the difference in the means by which they respectively reduce risk of future exploitation: vengeance motivates retaliatory behaviours while forgiveness motivates reparative behaviours. Hence to punish without vengeance and affective blame, but with forgiveness, can be cashed out as follows. Punishment is reconceived as a response to responsibility for wrongdoing which imposes consequences—no doubt typically negative but occasionally not, so long as they are serious and appropriate to the crime and the context—*that embody reparative strategies*. We not only foreswear any and all affective blame towards the offender as a person, but we employ reparative rather than retaliatory behaviours to fashion the consequences imposed by, and the environment and practices found within, the criminal justice system.

Within personal relationships between individuals, the extent to which we have clear and conscious choice over the decision to blame or to forgive is complex. On the one hand, forgiving is to some extent an act—something one can certainly refuse to do, or, alternatively, offer as a gift. On the other hand, it may sometimes seem as if no matter how hard one hopes or wills oneself to forgive, one cannot bring oneself to offer the proverbial olive branch—blame prevails, despite one’s best efforts. No doubt, there are methods to help us, as individuals, forgive as opposed to blame—if that is what we decide that we ought ideally to do.^51^ However, the criminal justice system is not an individual, but an institution. There is therefore ample scope for choice in how we punish within it, in that we can design practices and procedures and promote environments within the criminal justice system as a whole—in courts, prisons and the community—that are either blaming and retaliatory, or forgiving and reparative (for specific examples, see section 5).

Undoubtedly, implementation of such practices will require individuals who work within criminal justice institutions to perform tasks and accept responsibilities specific to their role that are designed to offer institutional forgiveness and promote reparation: if punishment is to proceed with forgiveness, that will affect the nature of criminal justice work. As with all professional roles, some individuals may then be more suited to the nature of the work than others. For instance, individuals who believe in forgiveness and reparation as a guiding ideal within criminal justice policy and practice, and who are skilled in modes of communication and interaction that are non-judgmental and non-aggressive, may find themselves better suited to the work than those whose outlooks and skills are different. But the design and implementation of these practices within the criminal justice system does not depend on individuals who work within it themselves *feeling* forgiveness at a personal level, but rather on their ability to perform their duties and abide by their professional roles. That is how there can be ample scope for choice in whether the criminal justice system, as an institution, is blaming or forgiving, and, correspondingly, in how it punishes, irrespective of the extent to which, as individuals, we have clear and conscious choice over the decision to blame or to forgive.

Choosing forgiveness and adapting the reparative behaviours identified by evolutionary psychology for use within the criminal justice system and as a form of punishment will not be simple or straightforward. In section 6, we begin the task of sketching what it might mean for the criminal justice system to punish with forgiveness in real terms. But we turn next to articulating some of the reasons we have to aspire to do so in theory.

## 5. From Instrumental to Ethical Reasons to Punish with Forgiveness

One of the more striking ideas to come from evolutionary psychology is that forgiveness—like vengeance—has evolved as a strategy to reduce risk of future harm in face of past exploitation. Indeed, when forgiveness works, it arguably offers a more reliable and economical risk reduction strategy than vengeance, because it does not depend on monitoring together with possessing and maintaining the power to retaliate. Long-term monitoring in contemporary contexts—whether in the form of incarceration in prisons, or in the form of supervision in the community—is both expensive and fallible. Costs are high, yet much offending goes undetected. Similarly, maintaining the appearance—let alone the reality—of having the power to detain and punish requires significant and sustained social and economic investment. In contrast, forgiveness—when it works—functions to reduce risk of future exploitation by fostering an intrinsic desire to end hostilities on the part of the exploiter—eliciting recognition of the value of the exploited party and an ongoing relationship with them to the exploiter. The success of forgiveness as a risk-reduction strategy does not therefore rely on monitoring and maintaining power.

The recent marked growth of the prison population and sentence lengths in many countries,^52^ together with the disturbing fact that rates of re-offending remain staggeringly high despite evidence that most persistent offenders desist over the longer term,^53^ suggest that the current criminal justice system, with its commitment to a mode of punishment that is retributive and expressive of affective blame, is not working to reduce re-offending rates.^54^ Indeed, vengeance as a risk reduction strategy is likely to harbour serious risks when implemented within contemporary socio-political and economic climates.^55^

Vengeance as a risk reduction strategy carries the inherent risk of creating a cycle of revenge. Indeed, the treatment offenders receive at the hands of the courts, inside prisons, and by parole services, can be debasing and humiliating.^56^ Such punishment is poised to create resentment and grudges among offenders so that—rather than reducing risk of re-offending—it may in fact contribute to bitterness towards ‘society’ and ‘authority’, bolstering an intrinsic desire to perpetuate hostilities. Moreover, this inherent risk to vengeance—namely, that it creates a cycle of revenge—may be compounded by the socio-politico-economic context in which the criminal justice system currently operates. Many offenders come from impoverished backgrounds of little opportunity and low socio-economic status. Many have suffered extreme forms of psycho-social adversity in childhood, including violence in the home, physical or sexual abuse and emotional neglect, bereavement and institutional care.^57^ Many are members of ethnic minorities who will have been the victims of discrimination and prejudice.^58^ Moreover adult substance misuse and mental health problems are associated with such childhood and systemic adversity.^59^ Such offenders are therefore already at the margins of society—deprived of many goods and opportunities that others possess, they are people whose early and later experiences may naturally make them feel outcast and uncared for—and so may for good reason believe that their relationships to others as well as to society at large have little proven value or benefit to them. Their mindset entering the criminal justice system may be that people are not to be trusted, and that society does not work in their favour. Retributive punishment that stigmatises and gives license to expressions of affective blame may therefore serve to further alienate such offenders from society—in effect, increasing the divide between ‘us’ and ‘them’ and shifting an already marginalised and underprivileged faction of our community into a *bona fide* out-group, thereby confirming their belief that there can be no valuable relationship between society and them—no mutual ‘fitness benefits’ in the language of evolutionary psychology. A hostile, anti-social attitude among offenders may as a result be entrenched by retributive, blaming modes of punishment enacted within contemporary socio-political and economic climates. If so, then vengeance as a risk reduction strategy may worsen the propensity for offending via its impact on the psychology of offenders, and so will depend for its effectiveness entirely and increasingly on monitoring and maintaining power.^60^

For wholly instrumental reasons—namely, the desire to reduce the risk of re-offending—we therefore have reason to question the value of retributive modes of punishment in contemporary contexts that stigmatise and give licence to expressions of affective blame. But the picture just painted of its potential effect on the structure of relationships within our society should also give us pause for ethical reasons. The basic political values of a broadly liberal society demand that respect and equality ideally accrue to all.^61^ These values are horrendously violated by strategies such as capital punishment, life imprisonment and post-sentence practices that carry enduring stigma—put otherwise, strategies such as killing, incapacitation through forced and permanent ostracisation and exclusion, and, finally, permanent shaming and branding^62^—which in effect endorse the treatment of offenders as out-group members. Rather, these values demand responses to crime that aim—even if they do not always succeed, and even if risk reduction and public protection are of course equally important values^63^—to restore offenders as full participant members of our society and repair the rupture to all that criminal offending creates. Vengeance and affective blame do not serve those values. Indeed, they may undermine them, by further marginalising and ostracising those who are already on the fringe of our society due to being victims of discrimination, inequality, and psychological, social and economic adversity.

In contrast with vengeance and affective blame, forgiveness may function to serve the basic political values of a broadly liberal society, while yet addressing the need to reduce the risk of re-offending. For it addresses this need by seeking to repair relations—by fostering in-group solidarity and mutually beneficial relationships as a means of overcoming hostilities and committing to future equality and respect for all parties involved. Given contemporary social, political and economic climates in countries such as Britain and the United States, together with a commitment to these values, we therefore have both instrumental and ethical reasons to consider working towards a reconception of punishment, as proceeding without vengeance and affective blame, but with forgiveness, so as to embody reparative strategies.^64^

We opened this article with a stark question: faced with wrongdoing, should we choose to blame and retaliate, or forgive and repair relations? We have argued that, from a theoretical perspective, the answer lies in forgiveness: we should choose to forgive and repair relations while yet holding offenders responsible and accountable for crime, by forgoing vengeance and affective blame in punishment. We do not, however, claim that this choice will always be easy to make let alone to effect in practice—even for those convinced of its correctness. Given that many offenders—even apart from their offending—may be viewed by the criminal justice system as well as society at large as low in Associational Value, replacing blame with forgiveness as a guiding ideal may require overcoming deep psychological as well as institutional barriers. What we hope to have established thus far is more theoretical, namely, that reconceiving punishment as proceeding with forgiveness represents an ideal towards which we have instrumental and ethical reasons to strive. In the next and final section, we begin the project of exploring what this reconception might mean in practice—not simply in theory—sketching how it could be realised at various stages of the criminal justice process, including conviction, sentencing, and the execution and longer-term effects of the sentence. Given the spectrum of real-world responses that exist to wrongdoing, how can we begin to move the criminal justice system further away from the extremes of blame and retaliation, and more towards the ideal of forgiveness and reparation?

## 6. Punishment with Forgiveness: Creating Institutions and Practices that ‘Wipe The Slate Clean’

Forgiveness may be a universal human adaptation, but expectations and practices connected to forgiveness are affected by cultural context. Before exploring what this reconception of punishment means in real terms, we need to draw attention to one element of our standard cultural expectations and practices connected to forgiveness which is potentially undermining to this project, namely, the belief that forgiveness *must be earned.*^65^ This belief expresses the view that forgiveness ought to be granted only after the offender has taken the initiative to apologise in full—they have repented, suffered and atoned. In other words, before we are willing to countenance forgiveness, we may be minded to ask offenders in effect to take the first step: to acknowledge without excuse or justification the entirety of their culpability and the nature of their offence, experience the full psychological weight of their wrongdoing, and accept punishment for it.

Forgiveness is a co-operative strategy that, to be optimal in reducing risk and repairing relations, requires both parties to participate. It is entirely appropriate to have expectations of offenders as part and parcel of a practice of forgiveness. However, these expectations must not smuggle in vengeance and affective blame under the guise of forgiveness. Asking offenders to earn forgiveness may in reality be an expression of retributive sentiment, manifesting a desire that wrongdoers feel debased and humiliated because of what they have done. If forgiveness is granted only after such sentiments have been indulged, then it comes too late to contribute to a reconception of punishment. Indeed, if such sentiments are indulged, they may—in keeping with vengeance—risk a cycle of revenge, where offenders are motivated to pay lip-service to apology, repentance and atonement, in order to reap the rewards of forgiveness, while yet harbouring bitterness and hostility. In other words, demanding that forgiveness is earned before it is offered may decrease the capacity of forgiveness to function effectively as a risk reduction strategy, by increasing the likelihood that offenders nurture hidden resentments and are not genuinely committed to refraining from future exploitation.

It may also decrease the availability of the kind of narrative told by *ex*-offenders of their journey to desistance. Based on extensive controlled studies of persistent offenders who ‘make good’—to use his term—and desist from crime, Maruna argues that ex-offenders employ ‘redemption scripts’ to make sense of their experience as a person who was once a criminal, but has now left that behind them.^66^ In our culture, such redemption scripts tend to posit a core self who was led astray by various forces and influences outside of their control. The offender eventually returns to their rightful path, partially because they had within them a ‘good self’ all along, partially through their own hard work, and partially through changed life circumstances and the help of others who believed in them. As a result of this experience, they are then in a position to ‘give back’ and offer help to others who find themselves also being led astray.^67^ In so doing, they gain a sense of purpose, and are able to reconcile themselves to their pasts. The redemption scripts told by those who have successfully desisted from crime tend to express strong belief in the current self’s powers of control and agency with respect to the present and the future. They do not, however, equally tend to acknowledge full control and agency with respect to past wrongdoing. Responsibility for past offending is not denied, but there are significant excuses, justifications and mitigating conditions that are an important part of the narrative. Crucially, this is essential to a narrative where the core self has always been to some extent ‘good’, but is—through various outside forces and influences—led astray.^68^

These studies suggest that asking offenders to acknowledge without excuse or justification the entirety of their culpability and the nature of their offence—in order to earn forgiveness—would not aid them in generating a redemption script. Rather, it is likely to further cement what Maruna calls a ‘condemnation script’ which portrays them as, by nature, criminal, deviant and outsiders—bad to the core, as we say. This, in turn, cannot but be destructive to the effectiveness of any reparative strategy, for it precludes offenders from believing in the possibility that they are the kind of person who could ever be of value to society and stand in mutually beneficial relations with others.

Hence, in exploring how punishment could proceed with forgiveness in practice—in designing real procedures and promoting environments within the criminal justice system that are genuinely reparative—we need to be alive to the way elements of our own cultural practice of forgiveness may undermine this task if we are not vigilant. It is an empirical question what in fact helps people to desist from crime and come to function as full participant members of our community. We may need to help current offenders tell a good redemption story in order to become ex-offenders, even though this requires us to forego satisfaction of our desire for full apology, repentance, suffering and atonement.

In light of this, we therefore suggest that we depart from this element of our standard forgiveness practice and begin by asking, not what offenders can do to earn our forgiveness, but *what the criminal justice system can do to encourage offenders to participate in a reparative process*. In other words, how can the criminal justice system in effect take the first step, offering forgiveness prior to it being earned?

As well as offering a clean—or at least cleaner—slate post-sentencing as discussed in section 2, no doubt part of this endeavour will require creating environments and practices at all stages of the criminal justice process that, in the most general of terms, disavow and discourage affective blame. This task is the upshot of the negative component of forgiveness identified in section 2, as *foreswearing* vengeance and affective blame in punishing. But we here wish to make some very preliminary suggestions, more specifically directed towards the particular question just mooted, of how the criminal justice system could—drawing on the insights of evolutionary psychology and the argument presented thus far—institutionalise forgiveness so as to encourage offender participation in reparative strategies.

Recall from section 3 that reparative behaviours include (at least) the following three types. First, the forgiver may communicate to the exploiter the extent of the damage they have suffered because of the exploitation. Second, the forgiver may indicate that, despite the damage, the possibility for a mutually beneficial future relationship exists if the exploiter commits to refraining from future exploitation—in effect, the forgiver will wipe the slate clean, if they can be convinced that the exploiter really will refrain from harming them further. Third, the forgiver may remind the exploiter of the mutually beneficial past relationship, in order to make salient the potential long-term costs to the exploiter of a permanent rupture to that relationship. How can these interpersonal behaviours be institutionally implemented with the criminal justice system? We distinguish three stages of the process—conviction, sentencing and the execution of the sentence—and suggest how each offers the opportunity to enact one of these strategies.

### A. Conviction

Conviction involves a formal declaration that a person is guilty of an offence by verdict of a jury or decision of a judge in a court of law: it is a judgment of responsibility for criminal behaviour. Its broader context, of course, is the trial, where the facts surrounding the offence are established. But within courtrooms which licence vengeance and affective blame, the process of trial and conviction can all too easily become a more generalised expression of ‘judgmentalism’ whereby the offender’s character is paraded, derided, and condemned.^69^ In other words, the courts may do more than judge a person as responsible for criminal behaviour—they may judge the person in themselves as ‘essentially’ of ‘bad character’.

This tendency of the trial and conviction stage of the criminal justice process to slide into character judgmentalism can undermine its potential to contribute to a redemption script. Rather than offering a process where offenders are invited to ‘own up’ to their conduct in a way that facilitates finding a path to a different future, they are required to ‘own up’ to the fact they are, in essence, bad to the core. When conviction becomes a form of condemnation of character rather than a declaration of responsibility for crime, its potential for reparation will be lost.

Rather, we suggest that to be reparative, trial and conviction must remain focused on the nature of the conduct, understood with respect to severity of offence and degree of responsibility, and its impact on others and the community—not turn to the nature of the offender as a person and their overall character. In effect, this stage of the criminal justice process can be used to communicate the extent of the damage suffered—the first reparative behaviour identified above. Such communication need not be blaming if conducted in a non-retaliatory and non-judgmental fashion. Instead, it can be a form of learning—helping the exploiter to recognise the true consequences of the exploitation and reflect on its meaning. Indeed, studies of restorative justice, which often emphasise direct communication of damage by the victim to the perpetrator, suggest that this can be one of the most important and meaningful aspects of the process to the perpetrator—if also, of course, emotionally difficult for them.^70^ Offenders may be straightforwardly unaware of some of the harm they perpetrate on others—whether because they have poor ability to predict or understand other people’s mental states or limited capacity for empathy, or because they repress or deny this truth, because of the pain, guilt, and shame that acknowledging it creates. If the trial and conviction stage of the criminal justice process is used to help offenders better recognise the nature of their actions and the effects on others and the community, it may aid psychological and moral growth, thereby serving reparative and rehabilitative ends. Conviction must not, however, be undertaken with affective blame if it is to have this effect—communication and learning is unlikely to be achieved if the trial and conviction process slides into one of character derision and condemnation, where offenders get their ‘noses rubbed’ in their wrongdoing.

### B. Sentencing

Upon conviction for an offence and in absence of diversion to hospital, the sentencing process then determines what punishment will be inflicted in response to the crime. In many jurisdictions, notably England and Wales and many of the US jurisdictions, sentencing must in practice proceed in accordance with established guidelines that constrain the type and degrees of punishment that can be imposed, relative to the level of responsibility for and severity of the crime. Typically, the sentence is decided by the judge and then received by the offender. As argued above, we believe some of these guidelines codify overly harsh and punitive sentencing practices. However, whether or not there is agreement on this point, there is yet scope to use this phase of the criminal justice process to actively encourage offenders to participate in a reparative process: rather than sentences being decided and delivered *de haut en bas*, the sentencing process could aim to create dialogue and discussion with offenders about the nature of the consequences—within, necessarily, the established sentencing framework that already exists—that might genuinely allow them to feel they had the opportunity for reparation and rehabilitation as a component of their sentence.^71^ Existing provisions in some systems for the deferral of sentence pending reparatory efforts on the offender’s part provide one potentially relevant institutional mechanism.^72^

Recall the second reparative strategy identified, which involves the forgiver indicating that, despite recognition of the damage, the possibility for a mutually beneficial future relationship exists if the exploiter commits to refraining from future exploitation. In effect, the forgiver will wipe the slate clean, if they can be convinced that the exploiter really will refrain from harming them further. Inviting offenders to say what, in their view, reparation and rehabilitation should involve—inviting them to engage in the process of fashioning and therefore also accepting the consequences to be imposed for their offending—might increase the possibility that these goods were realised. Such dialogue and discussion not only expresses respect and a willingness to interact and try to work together towards a productive relationship. By providing offenders with the opportunity to have a voice and role within sentencing, it also thereby allows them to begin the process of taking responsibility for their offence and in addition to consider what might realistically help them to repair the damage and move forward in a different way—to ‘make good’ in Maruna’s terms. In other words, using sentencing to create dialogue and discussion with offenders about the nature of the consequences appropriate to the offence, and how to make these reparative and rehabilitative, may increase their investment in and commitment to reparation and rehabilitation, and so too the likelihood that these goods are realised.

For clarity, we emphasise again that any such dialogue and discussion would in practice need to respect the sentencing guidelines relevant to the jurisdiction in question. But, equally, insofar as offenders appropriately identify, for example, particular kinds of public service or labour as offering the opportunity for a form of reparation, or therapy, workforce or educational training as offering an important personal pathway towards rehabilitation, these options need to be available to the courts to mandate as part of the sentence so long as they are concordant with guidelines. For any co-operative strategy to work and benefit both parties, both parties must be relied on to do their part. Offenders’ views of how their punishment might serve reparation and rehabilitation—when these are appropriate—must be heard and enacted.

### C. The Execution of the Sentence

In addition to the need for provision for adequate reparative and rehabilitative opportunities within both community and prison sentences, one further reform worth considering is to allow offenders to better maintain relationships with significant others—in a manner and to an extent which is very far from current practice within English and US prisons. When people go to prison, they often lose their relationships with their families and their friends. Undeniably, some of this loss is inevitable. But the fact that, in the UK and the US, many offenders are imprisoned very far from home, together with the severe restrictions on and monitoring of visits and phone calls, so that intimacy is infrequent if not outright impossible, cannot but raise the probability of interpersonal and social rupture. Such restrictions are neither humane nor necessary—as witnessed by the fact that a host of countries, including most prominently Canada, but also countries as diverse as Australia, Denmark, Spain, Israel and Saudi Arabia, allow conjugal or family visits of varying lengths of stay and frequency, as well as part-time imprisonment around continued employment.^73^

The final reparative behaviour identified above involves the forgiver reminding the exploiter of a mutually beneficial past relationship, in order to make salient the potential long-term costs to the exploiter of a permanent rupture to that relationship. In interpersonal contexts, forgivers seek to exploit the fact that they or their relationship has been of value to the exploiter, as a way of increasing the latter’s motivation to take the steps required to maintain it. Although no exact parallel is possible here between the criminal justice system and offenders (who, insofar as they have had a past relationship at all with the system, may not have had a good one), this strategy can yet be adapted if the criminal justice system provides a real opportunity for offenders to maintain—rather than lose—the people and relationships they care about in the community during their period of imprisonment. Imprisonment, of course, has terrible consequences for families and friends. Offenders may be strongly motivated to work towards desistence in order to protect these people and their relationships with them from these consequences. But this will not be possible if imprisonment permanently ruptures the relationships offenders care about most, isolating them even further. Hence, rather than criminal justice policies and practices that threaten and destroy these relationships, prisons—and, where relevant, community sentences—should instead aim to protect and maintain them as much as possible, as a way to furthering reparative and rehabilitative motivation in offenders.

These suggestions offer only a preliminary sketch of how criminal justice policy and procedures could be reformed in line with a reconception of punishment as proceeding with forgiveness. They are not all new, and there are of course many other suggestions that might be made. What we hope to have established is only how, if convinced of the merit of this reconception on both instrumental and ethical grounds, we can draw on evolutionary psychology to help us institutionalise forgiveness, by adapting interpersonal reparative behaviours to criminal justice institutions.

## 7. Conclusion

Designing a criminal justice system to take advantage of our evolutionarily endowed capacity to forgive and seek to repair relations must, inevitably, be a collaborative process between all of us—the institutions and officials comprising the criminal justice system, offenders and society at large, understood to include past and potential future victims. For only then will it be successful in reducing risk of re-offending and repairing relations. As well as holding responsible and to account, the system must offer an invitation to the offender to acknowledge their wrongdoing and help to fashion a sentence in a way that creates the opportunity to move forward in their lives—to be forgiven if they will do their part in making a better future for themselves and for others. The offender, in turn, must then step in to do the reparative and rehabilitative work involved in creating this different future. Acknowledgement of past wrongdoing and commitment to active change is no doubt central to this process. And, in all likelihood, for many offenders, this will provoke feelings of guilt, regret and remorse, as they face up to and make sense of the damage their offending has done to others as well as its role in their own lives. But in tandem with holding responsible and to account, reparative strategies can be offered by the criminal justice system and society, without requiring offenders first to earn them. Rather, we can ourselves take the initial step, and look forwards rather than backwards, and choose to punish not with vengeance and affective blame, but with forgiveness.

## Footnotes

^1^ For discussion, see D Garland, *The Culture of Control* (OUP 2001) 139–65. Victoria McGeer argues that, although blame and the demand for vengeance may be a basic human response to wrongdoing, retribution in itself does not bring the expected feelings of vindication; hence the propensity for escalating calls for retribution if this response is not tempered by regulating principles and practices (V McGeer, ‘Civilising Blame’ in DJ Coates and NA Tognazzini (eds), *Blame: Its Nature and Norms *(OUP 2013); for a review of some of the empirical evidence supporting McGeer’s position, see F Funk, V McGeer and M Gollwitzer, ‘Get the Message: Punishment Is Satisfying If the Transgressor Responds to its Communicative Intent’ 40 (2014) Pers and Soc Psych Bull 986).

^2^ For an extraordinary story of forgiveness, listen to ‘Dear Hector’ (*RadioLab* <www.radiolab.org/story/317629-dear-hector/> accessed 28 July 2014) where Hector Black, a man who forgave and ultimately befriended his daughter’s rapist and murderer, is interviewed; another remarkable example is the story of prisoner of war Eric Lomax, recounted in his autobiography *The Railway Man *(Jonathan Cape 1995).

^3^ See section 4.

^4^ See for example A von Hirsch, *Doing Justice: The Choice of Punishments *(Hill and Wang 1976) and *Censure and Sanctions* (Clarendon Press 1993); MS Moore, *Placing Blame: A General Theory of the Criminal Law* (Clarendon Press 1997); D McDermott, ‘The Permissibility of Punishment’ (2001) 20 L Phil 403.

^5^ The amount of blameworthiness is understood as a combination of degree of responsibility of the offender and severity of the offence. In N Lacey and H Pickard, ‘The Chimera of Proportionality: Institutionalising Limits on Punishment in Contemporary Social and Political Systems’ (2015) 78 MLR 216, we argue that in contemporary contexts, proportionality is in fact nothing more than an abstract ideal which in itself is powerless to meaningfully limit punishment in the way suggested by the ‘justice’ model.

^6^ There are of course important differences between ‘just deserts’ theorists. But even Antony Duff, who is explicitly committed to promoting reform and reconciliation, insists that hard treatment is intrinsic to the communicative theory of punishment, and that mercy is extrinsic (RA Duff, *Punishment, Communication and Community* (OUP 2001)). Our argument is that the commitment to hard treatment as a defining feature of punishment risks, in practice, undermining the aspiration to foster inclusiveness by inviting exclusionary punitiveness (see sections 5 and 6; cf M Matravers, ‘Duff on Hard Treatment’ in R Cruft, MH Kramer, MR Reiff (eds), *Crime, Punishment and Responsibility: The Jurisprudence of Antony Duff* (OUP 2011); and J Tasioulas, ‘Where is the Love? The Topography of Mercy’ in Cruft, Kramer and Reiff, ibid.). While von Hirsch’s theory does not assume that punishment must involve hard treatment, the idea of ‘censure’ to which he appeals both attaches more naturally to persons than to conduct, and evokes the flavour of blame while lacking any explicit renunciation of its appropriateness (von Hirsch, *Censure and Sanctions* (n 4)). Other recent contributions to penal philosophy in the broadly retributive tradition have explicitly embraced the idea that punishment is intrinsically exclusionary or stigmatising. For example, in a recent paper Douglas Husak argues that ‘a state response to conduct does not qualify as punitive unless it is designed to censure *and to stigmatize*’ (D Husak, ‘Lifting the Cloak: Preventive Detention as Punishment’ (2011) 48 San Diego L Rev 1173, 1182 (our emphasis)). And Daniel McDermott argues for the essentially exclusionary dynamic of retribution, and regards imprisonment as a presumptively acceptable penalty on the basis that, in the style of banishment, it excludes wrongdoers from the moral community (D McDermott, ‘The Permissibility of Punishment’ (2001) 20 L Phil 403). Note the resonance between these views and the discussion of vengeance arising from evolutionary psychology, particularly the risk that it treats offenders not as members of our society but as a bona fide out-group; see sections 4 and 5. For further discussion of the link between the ‘justice’ model and blame, see N Lacey and H Pickard, ‘From the Consulting Room to the Court Room? Taking the Clinical Model of Responsibility Without Blame into the Legal Realm’ (2013) 33 OJLS 1.

^7^ Note that the link between punishment and the imposition of retaliatory costs—never mind the desire that the other suffer in turn—is not an essential part of the concept. For a familiar example of a non-retaliatory, non-vengeful practice of punishment, think of how a loving parent may punish a child who has done wrong.

^8^ Note too that our pragmatism resonates with the literature on restorative justice exploring the potential compatability between restorative and retributive justice, in theory and practice. See eg A von Hirsch and others (eds), *Restorative Justice and Criminal Justice: Competing or Reconcilable Paradigms?* (Hart Publishing 2004); S Garvey, ‘Restorative Justice, Punishment and Atonement’ (2003) Utah L Rev 303; RA Duff, ‘Restorative Punishment and Punitive Restoration’ in L Walgrave (ed), *Restorative Justice and the Law* (Willan Publishing 2002); and L Zedner, ‘Reparation and Retribution: Are they Reconcilable?’ (1994) 57 MLR 228.

^9^ Lacey and Pickard, ‘From the Consulting Room’ (n 6).

^10^ See Lacey and Pickard, ‘From the Consulting Room’ (n 6). For a detailed discussion of the clinical model itself, see H Pickard, ‘Responsibility Without Blame: Empathy and the Effective Treatment of Personality Disorder’ (2011) 18 Philosophy, Psychiatry, Psychology 209 and ‘Responsibility Without Blame: Philosophical Reflections on Clinical Practice’ in KWM Fulford and others (eds), *The Oxford Handbook of Philosophy and Psychiatry *(OUP 2013).

^11^ For the classic articulation of this link, see PF Strawson, ‘Freedom and Resentment’ (1962) 48 Proceedings of the British Academy 1.

^12^ See G Watson, ‘Responsibility and the Limits of Evil’ in G Watson (ed), *Agency and Answerability: Selected Essays* (OUP 2004) 220. Note that Watson himself does not endorse this view.

^13^ See for example M Zimmerman, *An Essay on Moral Responsibility *(Roman and Littlefield 1988); RJ Wallace, *Responsibility and the Moral Sentiments* (Harvard University Press 1994); JM Fischer and M Ravizza, *Responsibility and Control: A Theory of Moral Responsibility* (CUP 1998).

^14^ For further discussion of affective blame see H Pickard, ‘Irrational Blame’ (2013) 74 Analysis 613.

^15^ For discussion see eg J Murphy, *Punishment and the Moral Emotions* (OUP 2012); Duff, *Punishment, Communication and Community* (n 6) 82–99; JM Fischer, ‘Desert and the Justification of Punishment’ in TA Nadelhoffer (ed), *The Future of Punishment* (OUP 2013).

^16^ For further discussion see S Pearce and H Pickard, ‘Finding the Will to Recover: Philosophical Perspectives on Agency and The Sick Role’ (2005) 36 J Med Ethics 831; Pickard, ‘Responsibility Without Blame: Empathy’ (n 10); Pickard, ‘Responsibility Without Blame: Philosophical Reflections’ (n 10); S Pearce and H Pickard, ‘How Therapeutic Communities Work: Specific Factors Related to Positive Outcome’ (2012) 59 Int’l J Soc Psych 636; H Pickard, ‘Psychopathology and the Ability to do Otherwise’ (2015) 90 Phil & Phenomenological Research 1135; H Pickard, ‘Responsibility Without Blame: Therapy, Philosophy, Law’ (2014) Prison Service J 10.

^17^ For further discussion of the distinction between ‘detached’ and ‘affective’ blame, see H Pickard, ‘Responsibility Without Blame: Empathy’ (n 10); H Pickard, ‘Responsibility Without Blame: Philosophical Reflections’ (n 10); H Pickard, ‘Irrational Blame’ (n 14); Lacey and Pickard, ‘From the Consulting Room’ (n 6). For related discussion of the role of morality within psychiatric practice, see H Pickard, ‘Scrupulosity and the Shady Morality of Psychiatry’ in D Moseley and G Gala (eds), *Philosophy and Psychiatry: Problems, Intersections and New Perspectives* (Routledge forthcoming).

^18^ For discussion of the differences and hence the corresponding limitations of the analogy, see Lacey and Pickard ‘From the Consulting Room’ (n 6).

^19^ Or to put this in language reflecting those aspects of the ‘justice’ model that we reject—the need for punishment to be ‘proportional’ to degree of ‘blameworthiness’ (see n 5).

^20^ In our society, forgiveness—alongside these other sentiments—also has religious overtones. Although we acknowledge the religious underpinnings of some of the philosophical and legal writings we draw on, our aim in this and the next section is to offer an account of forgiveness which is secular, in that it can be understood and institutionalised within the criminal justice system wholly apart from religious symbols, beliefs, and practices.

^21^ See JG Murphy and J Hampton, *Forgiveness and Mercy (*Cambridge Studies in Philosophy and Law, CUP 1988); C Steiker, ‘Tempering or Tampering? Mercy and the Administration of Criminal Justice’ in A Sarat and N Hussain (eds), *Forgiveness, Mercy and Clemency* (Stanford University Press 2007); and Tasioulas (n 6).

^22^ The phrase ‘make good’ is borrowed from S Maruna, *Making Good: How Ex-convicts Reform and Rebuild their Lives* (American Psychological Association 2001). For further discussion see section 6; see too M Wright, *Making Good: Prisons, Punishment and Beyond* (2^nd^ edn, Waterside Press 2008).

^23^ I Kant, ‘Metaphysics of Morals’ in *Kant: Political Writings *(Cambridge Texts in the History of Political Thought, CUP 1991) 142.

^24^ See L Allais, ‘Wiping the Slate Clean: The Heart of Forgiveness’ (2008) 36 P&PA 33; also J Murphy, ‘Forgiveness, Reconciliation, and Responding to Evil: A Philosophical Overview’ and ‘Christian Love and Criminal Punishment’ in his *Punishment and the Moral Emotions: Essays in Law, Morality, and Religion *(OUP 2012).

^25^ For this reason, it is a natural part of early modern conceptions and rituals of punishment, where rulers were invested with a sacred or divine form of authority, allowing them to exercise mercy or pardon at will, without concern for consistency or precedent. For discussion of these themes, see Lacey and Pickard, ‘The Chimera of Proportionality’ (n 5).

^26^ See references at n 21; and also J Murphy, ‘Repentence, Mercy, and Communicative Punishment’ and ‘Remorse, Apology, and Mercy’ in his *Punishment and the Moral Emotions *(n 24).

^27^ The breadth of crime as a category of course implies that it is impossible to generalise about the demographics of offending; but it is clear that the prison population in most countries exhibits significant levels of social disadvantage. See eg, R Allen and others, *A Presumption Against Imprisonment* (British Academy 2014) part I, 26–52.

^28^ For further discussion of the increase in prison population and sentence length as a challenge to the ‘justice’ model, see Lacey and Pickard, ‘The Chimera of Proportionality’ (n 5).

^29^ Pardons are acts as opposed to emotions or reactive attitudes, but they nonetheless possess some similarities both to mercy and forgiveness. Like mercy, pardons are exercised *de haut en bas* and typically serve to reduce or remove punishment. Like forgiveness, they may be enacted in order to promote reparative ends.

^30^ Allais, ‘Wiping the Slate Clean’ (n 24).

^31^ See J Butler, ‘Upon Forgiveness of Injuries (sermon IX)’ in WE Gladstone (ed), *Sermons of Joseph Butler* (1^st^ edn, Bishop Durham 1897) 127–41; D Novitz, ‘Forgiveness and Self-respect’ (1988) 58 Phil & Phenomenological Research 299; Murphy and Hampton (n 21); RC Roberts, ‘Forgivingness’ (1955) 32 Am Phil Q 289; P Hieronymi, ‘Articulating an Uncompromising Forgiveness’ (2001) 62 Phil & Phenomenological Research 529; J Murphy, *Punishment and the Moral Emotions* (n 24).

^32^ Allais, ‘Wiping the Slate Clean’ (n 24).

^33^ Allais also emphasises how her account explains how one can forgive those, such as family, friends and intimates, for grievances that one held against them, despite the fact that one never failed to treat them with goodwill or hold them in positive regard as a person.

^34^ As should be apparent, we also depart from the more retributive strand that forms part of her discussion, nuanced as it is. See too L Allais, ‘Restorative Justice, Retributive Justice, and the South African Truth and Reconciliations Commission’ (2011) 39 P&PA 331.

^35^ See G Pettigrove, *Forgiveness and Love *(OUP 2012). Importantly, we can still allow that the victim of the offence has a special relationship to the offender such that their forgiveness may matter in a different way and to a different extent. For instance, it may be that all other acts of forgiveness feel hollow to the offender unless and until the victim forgives. Alternatively, third parties may be reluctant to forgive unless the victim too is willing. But recognition of a special role for victim forgiveness does not require it to be the case that only victims have the power to forgive. Insofar as culpable offending engenders hostile, negative emotions and attitudes towards the offender—whether in third parties or in offenders themselves—they too are capable of forgiving the offence, by forbearing from allowing it to colour their emotions and attitudes towards the offender as a person. See L Bovens, ‘Must I be Forgiven?’ (2009) 69 Analysis 227 for a discussion of *offenders’ distress *when victims refuse to forgive.

^36^ Roberts (n 31).

^37^ See eg J Braithwaite, *Crime, Shame, and Reintegration* (CUP 1989); J Braithwaite, *Restorative Justice and Responsive Regulation* (OUP 2002); L Sherman and H Strang, *Restorative Justice: The Evidence* (The Smith Institute 2007); G Robinson and J Shapland, ‘Reducing Recidivism: A Task for Restorative Justice?’ (2008) 48 British J Criminology 337; J Shapland, G Robinson and A Sorsby, *Restorative Justice in Practice: Evaluating What Works for Victims and Offenders* (Routledge 2011); M Rossner, *Just Emotions: Rituals of Restorative Justice* (OUP 2013).

^38^ For clarity, note that we do not claim that the law forgives the offence *for the victim *or *on behalf of the victim*—that power lies only with the victim—but rather in its own right, as a third-party with institutional responsibility for enforcing the criminal law.

^39^ See D Garland, ‘Penality and the Penal State’ (2013) 51 Criminology 475; J Manza and C Uggen, *Locked Out: Felon Disenfranchisement and American Democracy* (OUP 2006).

^40^ Duff, *Punishment, Communication and Community* (n 6)*. *See references in n 6 for further discussion.

^41^ Although we draw on evolutionary psychology as a source of fresh insight, it is important to be clear that we do not mean to commit ourselves to the precise details of any particular account of its nature and explanatory scope: a degree of epistemological scepticism about (some aspects or uses of) evolutionary psychology is compatible with nonetheless holding that the subject can offer a valuable perspective. For discussion of such scepticism see D Buller, *Adapting Minds: Evolutionary Psychology and the Persistent Quest for Human Nature *(MIT Press 2005), K Sterelny, *Thought in a Hostile World: The Evolution of Human Cognition* (Wiley-Blackwell 2003); and H Rose and S Rose, *Alas Poor Darwin: Arguments Against Evolutionary Psychology *(Random House 2010). Equally, there are important questions about how adaptations that have evolved due to their fitness benefits in small-scale early human communities function in large-scale modern societies, and, correspondingly, how we can construct modern societies so that our evolutionary inheritance best serves our contemporary values. For discussion of some of the large-scale socio-political and economic factors that may be relevant to fostering solidarity between citizens and promoting forgiveness, see Lacey and Pickard, ‘The Chimera of Proportionality’ (n 5).

^42^ M McCullough, R Kurzban and B Tabak, ‘Cognitive Systems for Revenge and Forgiveness’ (2013) 36 Behavioral and Brain Sciences 1. See also MB Petersen and others, ‘Evolutionary Psychology and Criminal Justice: A Recalibrational Theory of Punishment and Reconciliation’ in H Høgh-Olesen (ed), *Human Morality and Sociality: Evolutionary and Comparative Perspectives (*Palgrave MacMillan 2010); MB Petersen and others, ‘To Punish or Repair? Evolutionary Psychology and Lay Intuitions about Modern Criminal Justice’ (2012) 33 Evolution and Human Behaviour 682.

^43^ There is of course the potential extrinsic cost that other members of the exploiter’s group may then try to kill or permanently incapacitate us in turn, and so exploitation and hostile interactions between groups are prolonged. See below in relation to the risks of vengeance as a strategy for protecting against risk of future exploitation within groups.

^44^ The evolutionary psychology literature typically elaborates this claim in terms of distinctive forms of Welfare Trade-off Ratios (WTRs). WTRs are conceptualised as internal regulatory variables that are continuously updated on the basis of new information and function to guide social decision-making. They reflect how much an individual values another’s welfare in relation to their own—put crudely, how much they are willing to ‘trade’ these ‘off’ against one another—and so guide decision-making with respect to what amount of cost an individual is willing to impose on another in order to gain what amount of benefit. WTRs are posited as coming in both ‘monitored’ and ‘intrinsic’ forms which are associated with vengeance versus forgiveness strategies respectively. For the purposes of our argument in this paper, the essential points we wish to glean from the evolutionary psychological literature can be captured without this theoretical commitment, useful as it may be as a model of social decision-making.

^45^ McCullough, Kurzban and Tabak (n 42).

^46^ ibid.

^47^ ibid.

^48^ Petersen and others, ‘Evolutionary Psychology’ (n 42) 110 ff; Petersen and others, ‘To Punish or Repair?’ (n 42). See also McCullough, Kurzban and Tabak (n 42). For similar findings from social psychology, see M Wenzel and others, ‘Retributive and Restorative Justice’ (2008) 32 L Human Behaviour 375; and M Wenzel and I Thielmann, ‘Why we Punish in the Name of Justice: Just Desert Versus Value Restoration and the Role of Social Identity’ (2006) 19 Social Justice Research 450. In Lacey and Pickard, ‘The Chimera of Proportionality’ (n 5), we argue that the orientation towards reparation or retaliation may be associated not only with features of the exploiter and their relationship with the group, but also with broader features of the socio-political and economic context.

^49^ Petersen and others, ‘To Punish or Repair?’ (n 42).

^50^ Note that, in apparent opposition to this claim, PH Robinson and JM Darley, ‘Intuitions of Justice: Implications for Criminal Law and Justice Policy’ (2007) 81 S Cal L Rev 1, have argued that the desire to punish is innate and universal in response to severe offending. However, the studies they cite in support of their argument do not offer participants a choice between a retaliatory and a reparative/rehabilitative response, but rather a choice between retaliation or no response, or between degrees of retaliatory response. We do not dispute that vengeance is a universal human adaptation, nor that it can be an automatic response. Rather, we dispute the assumption that it is the only possible universal human adaptation, and that its ubiquity—together with its automaticity—is evidence that it is wholly innate as opposed to partially culturally mediated or learned. For further discussion, see section 5.

^51^ For a review and analysis of clinical interventions that promote forgiveness, see NG Wade and EL Worthington, ‘In Search of a Common Core: A Content Analysis of Interventions to Promote Forgiveness’ (2005) 42 Psychotherapy: Theory, Research, Practice 160.

^52^ See N Lacey, *The Prisoners’ Dilemma: Punishment and Political Economy in Contemporary Democracies* (CUP 2008).

^53^ Maruna (n 22).

^54^ Studies from the US and Canada suggest that, regardless of the severity of the initial offence, adolescents who are imprisoned in response to an offence are significantly more likely to be re-imprisoned as adults, compared with adolescents who commit similar offences but are not sent to prison. See A Aizer and J Doyle Jr, ‘Juvenile Incarceration and Adult Outcomes: Evidence from Randomly-assigned Judges’ (National Bureau of Economic Research 2011) and U Gatti, RE Tremblay, and F Vitaro, ‘Iatrogenic Effect of Juvenile Justice’ (2009) 50 J Child Psychology and Psychiatry 991. For a moving account of how juvenile detention centres in Spain are fashioned to offer care and effectively promote learning, growth, and rehabilitation, as opposed to being modelled on more standard, adult prisons, see E Allison and S Hattenstone, ‘Tough Love: Is This a Model Prison for Children?’ *The Guardian* (London, 14 November 2014) <www.theguardian.com/society/2014/nov/07/tough-love-young-prisoners-spain-model-prison> accessed 23 February 2015.

^55^ For further discussion of the importance of socio-political and economic context to the form and impact of punishment, see Lacey and Pickard, ‘The Chimera of Proportionality’ (n 5).

^56^ A Liebling, ‘Moral Performance, Inhuman Treatment and Degrading Treatment and Prison Pain’ (2011) 13 Punishment and Society 530; A Liebling, assisted by H Arnold, *Prisons and their Moral Performance: A Study of Values, Quality and Prison Life *(Clarendon Studies in Criminology, OUP 2004); A Liebling and S Maruna (eds), *The Effects of Imprisonment* (Willan Publishing 2005); J Sim, *Punishment and Prisons: Power and the Carceral *(Sage 2009). For a telling glimpse of the world of a busy Chicago criminal court, see S Bogira, *Courtroom 302: A Year Behind the Scenes in an American Criminal Courthouse* (Vintage 2005).

^57^ J Peay, ‘Mentally Disordered Offenders, Mental Health and Crime’ in M Maguire, R Morgan and R Reiner (eds), *The Oxford Handbook of Criminology *(5^th^ edn, OUP 2012); R Allen and others, *A Presumption Against Imprisonment *(n 27) part I, para 31.

^58^ See C Phillips and B Bowling, ‘Ethnicities, Race, Crime and Criminal Justice’ in Maguire, Morgan and Reiner (n 57).

^59^ For an excellent review of much of the relevant data on substance misuse, see G Heyman, *Addiction: A Disorder of Choice *(Harvard University Press 2009). For more general associations between childhood and systemic adversity and mental health see CS Aneshenshel and JC Phelan (eds), *Handbook of the Sociology of Mental Health* (Springer 2006).

^60^ We note, but do not here address, the very important question of whether the background conditions of extreme adversity and socio-economic inequality just noted impact on our right to hold responsible and punish—let alone affectively blame—those in our society who have suffered them. Arguably, the moral standing to hold to account is premised on relatively equal relationships, and so may be undermined in radically unequal societies such as our own: when background conditions are unjust, the legitimacy of the criminal law to convict and sentence may be compromised. See Lacey and Pickard, ‘From the Consulting Room’ (n 6); J Braithwaite and P Pettit, *Not Just Deserts: A Republican Theory of Criminal Justice* (Clarendon Press 1990) 196–200; Duff, *Punishment, Communication and Community* (n 6) 175–201.

^61^ The classic statement of this liberal position is to be found in the work of Ronald Dworkin; see for example *Taking Rights Seriously* (Duckworth 1977) 180–83, 272–78 and *A Matter of Principle* (Harvard University Press 1985).

^62^ For discussion see Lacey and Pickard, ‘From the Consulting Room’ (n 6).

^63^ Research on psychopathy suggests that some individuals may have a reduced capacity for pro-social relationships and behaviour (for a state-of-the art review, see A Raine, *The Anatomy of Violence* (Penguin 2013)). On the other hand, the majority of offenders are not psychopathic and eventually desist from crime (Maruna (n 22)). Moreover, there are promising new treatment developments for adolescents with psychopathic tendencies—which, strikingly, achieve results through rewarding good behaviour, rather than punishing bad behaviour—although much more research needs to be conducted (MF Caldwell, ‘Treatment of Adolescents with Psychopathic Features’ in K Kiehl and W Sinnott-Armstrong (eds), *Handbook of Psychopathy and Law* (OUP 2013)). While the problems of accurate prediction are notorious (for a review of evidence, see A Ashworth and L Zedner, *Preventive Justice *(OUP 2014) ch 6), we leave open the possibility that there may be some individuals, with or without diagnosed psychopathic tendencies, where the risk of re-offending is so high that reparative and rehabilitative strategies—as opposed to incapacitating strategies—cannot at present be justified. However, the basic political values at stake, namely, that respect and equality ideally accrue to all, suggest that, even in such extreme cases, we ought to remain open to the possibility that reparation and rehabilitation may be possible in future, if such evidence presents itself. Hence, even when the risk of offering forgiveness and the chance for reparation cannot be justified at present, we can remain committed to these values by retaining a willingness to reconsider this decision in future if adequate reason is found, rather than adopting an attitude of ‘locking up and throwing away the key’. Relatedly, there can be no doubt that adequate mental health screening and ongoing treatment is an extremely important component of a just and effective criminal justice process (see J Peay, *Mental Health and Crime* (Routledge 2011)).

^64^ In England and Wales, there is also legal reason to embrace this reconception. The purposes of sentencing encoded in the Criminal Justice Act 2003, s 142, include not only punishment, but reduction of crime, reform and rehabilitation, public protection, and the making of reparation by the offender to those affected by the offence. Insofar as the reconception of punishment as proceeding with forgiveness that we advocate serves to unite and further these multiple aims, it better underpins and promotes English law. For further discussion of the role of considering the purposes of sentencing in fashioning criminal justice institutions and practices that promote responsibility without blame, see Lacey and Pickard, ‘From the Consulting Room’ (n 6).

^65^ A Lazare, *On Apology* (OUP 2004); see also Hieronymi (n 31); L Bovens, ‘XII-Apologies’ (2008) 108 Proceedings of the Aristotelian Society 219; Bovens, ‘Must I be Forgiven?’ (n 35); and A Martin, ‘Owning Up and Lowering Down: The Power of Apology’ (2010) 107 J Phil 1. Martha Nussbaum offers an extensive discussion of the genealogy of this aspect of forgiveness, which she calls ‘transactional forgiveness’, within both Jewish and Christian traditions, and a correlated argument against the appositeness of forgiveness thus understood in contemporary contexts. See Martha Nussbaum, *Anger and Forgiveness: Resentment, Generosity, Justice *(forthcoming) chs 3 and 4. Nussbaum’s conception of forgiveness does not draw on evolutionary psychology and hence differs significantly from the account we have offered here, which, despite some crucial differences, bears some similarity to her conception of unconditional generosity and love. Note too that the idea that the offender must earn victim forgiveness is a component of some restorative justice theory and practice. For discussion, see references in n 37.

^66^ Maruna (n 22)*.*

^67^ For example, many ex-offenders are able to find productive, satisfying work as youth support workers and counsellors. Indeed, there is ample evidence that ‘giving back’ not only contributes to desistance but is also effective: people listen to peers or others who have ‘been there’ differently from the way they listen to those in authority and who have not had the same experience. See Maruna (n 22); also Pearce and Pickard, ‘How Therapeutic Communities Work’ (n 16).

^68^ See too S Maruna and RE Mann, ‘A Fundamental Attribution Error? Rethinking Cognitive Distortions’ (2006) 11 The British Psychological Society 155. Note however that there may be different forms of redemption script, in part depending on the culture and context in which they are created. Alisa Stevens argues that Therapeutic Community prisons are distinctive in enabling offenders to reconstruct their identity so a ‘new’ and ‘better’ self emerges—thereby engendering less reliance on a core self that was ‘good’ all along, at least to some extent (A Stevens, ‘“I am the Person now I was Always Meant to be”: Identity Reconstruction and Narrative Reframing in Therapeutic Community Prisons’ (2012) 12 Criminology and Criminal Justice 527; see too her book *Offender Rehabilitation and Therapeutic Communities: Enabling Change the TC Way *(Routledge 2012). For a study of how clinicians who work with sex offenders may have a detrimental effect on recidivism rates by insisting on retrospective responsibility for past wrongdoing as opposed to focusing on prospective responsibility for future behaviour, see J Ware and RE Mann, ‘How Should “Acceptance of Responsibility” be Addressed in Sexual Offending Treatment Programs?’ (2012) 17 Aggression and Violent Behavior 279.

^69^ N Lacey, ‘The Resurgence of Character: Criminal Responsibility in the Context of Criminalisation’ in RA Duff and S Green (eds), *Philosophical Foundations of Criminal Law* (OUP 2011); see too G Watson, ‘Standing in Judgment’ in DJ Coates and NA Tognazzini (eds), *Blame: Its Nature and Norms *(OUP 2013).

^70^ See references at n 37. If appropriately managed, this process can also be healing—if equally emotionally difficult—for victims, in allowing them to have a voice and occasion to express the impact of the offence on them and the damage suffered. Note for emphasis that, on the reconception of punishment we are advocating, the claims of victims to recognition of and justice for wrongs suffered is robustly upheld, through the imposition of consequences—typically negative, always serious and appropriate—in response to responsibility for wrongdoing, together with the focus on fashioning criminal justice policies and practices that genuinely work to reduce risk of re-offending.

^71^ Some restorative justice processes allow for precisely such dialogue and discussion: see references at n 37. Intriguingly, Brent Fisse and John Braithwaite argue for a similar approach in response to corporate crime, whereby courts and corporations are asked to work together to determine appropriate forms of corporate accountability, including a demand for review of relevant practices to ensure better practice in future and where appropriate making reparation. See B Fisse and J Braithwaite, *Corporations, Crime and Accountability* (CUP 1993).

^72^ See eg Criminal Justice Act 2003, sch 23.

^73^ For discussion of relevant practices in the criminal justice system in Nordic countries, see eg J Pratt and A Eriksson, *Contrasts in Punishment: An Explanation of Anglophone Excess and Nordic Exceptionalism* (Routledge 2013) ch 1.

